# Pulsed Electric Field Ablation of Epicardial Autonomic Ganglia: Computer Analysis of Monopolar Electric Field across the Tissues Involved

**DOI:** 10.3390/bioengineering9120731

**Published:** 2022-11-27

**Authors:** Ana González-Suárez, Barry O’Brien, Martin O’Halloran, Adnan Elahi

**Affiliations:** 1 School of Engineering, University of Galway, H91 TK33 Galway, Ireland; 2 Translational Medical Device Lab, University of Galway, H91 YR71 Galway, Ireland; 3 AtriAN Medical Limited, Unit 204, NUIG Business Innovation Centre, Upper Newcastle, H91 W60E Galway, Ireland

**Keywords:** cardiac arrhythmia, computer modelling, epicardial ablation, ganglionated plexi, pulsed field ablation

## Abstract

Background and objectives: Pulsed Electric Field (PEF) ablation has been proposed as a non-thermal energy to treat atrial fibrillation (AF) by epicardial ablation of ganglionated plexi (GP), which are embedded within epicardial fat. Our objective was to study the distribution of the electric field through the involved tissues (fat, GPs, myocardium and blood) during epicardial PEF ablation. Methods: A two-dimensional model was built considering different tissue layers below the ablation device which consists of an irrigated electrode. The 1000 V/cm threshold was used to estimate the ‘PEF-zone’. Results: The PEF-zone was almost 100% circumscribed in the epicardial fat layer, with very little incidence in the myocardium. The presence of the saline on the epicardial fat causes the PEF-zone to spread laterally around the electrode from ~5 mm to ~15 mm, relatively independently of how embedded the electrode is in the saline layer. For a saline layer well spread over the tissue surface and an electrode fully embedded in the saline layer, the PEF-zone width decreases as the fat layer thickens: from ~15 mm for fat thickness of 1 and 2 mm, down to ~10 mm for fat thickness of 5 mm. The presence of a GP in the center of the fat layer hardly affects the size of the PEF-zone, but significantly alters the distribution of the electric field around the GP, resulting in progressively lower values than in the surrounding adipose tissue as the fat layer thickness increased. Conclusions: Our results suggest how some procedural (irrigation) and anatomical parameters (fat thicknesses and presence of GPs) could be relevant in terms of the size of the tissue area affected by pulsed field ablation.

## 1. Introduction

Pulsed Electric Field (PEF) ablation, also known as Pulsed Field Ablation (PFA), has been recently used to ablate cardiac ganglionic plexi (GP), as the contribution of the autonomic nervous system to the induction and maintenance of atrial fibrillation (AF) is increasingly appreciated [1]. The purpose of this technique is to destroy GPs, which are embedded in epicardial fat [2,3,4]. This is done by placing a multi-electrode ablation catheter on the epicardial surface. In a previous computer modelling study incorporating the real anatomy of the heart and the patient’s torso, we found that the electric field distribution is mainly limited to the vicinity of the electrodes and is practically negligible in adjacent organs (oesophagus and lungs). This confirmed that the limited-domain models offer similar results than full-torso models in terms of lesion size, reducing the complexity of the modelling [5]. However, this computational model did not include the detailed geometric structure of the heart (epicardial fat, GPs, myocardium and blood), nor the effect of the saline infusion. To date, computer modelling has been mainly used to study endocardial PFA (i.e., where the ablation electrodes are immersed in the cardiac chamber, surrounded by blood and placed on the endocardium solely on the area to be ablated) [6,7,8]. Relevantly, the study by Verma et al. [6] suggested that the presence of tissues with very different electrical conductivity caused large electric field gradients. Bearing this in mind and taking into account the great disparity in the electrical conductivity of the tissues involved in the epicardial ablation (fat, GPs, myocardium and blood), we planned a computer modelling study to assess how the electric field is distributed across these tissues, and consequently, under what conditions can epicardial PEF ablation of GPs be more effective.

## 2. Methods

### 2.1. Model Geometry

**Figure 1:**
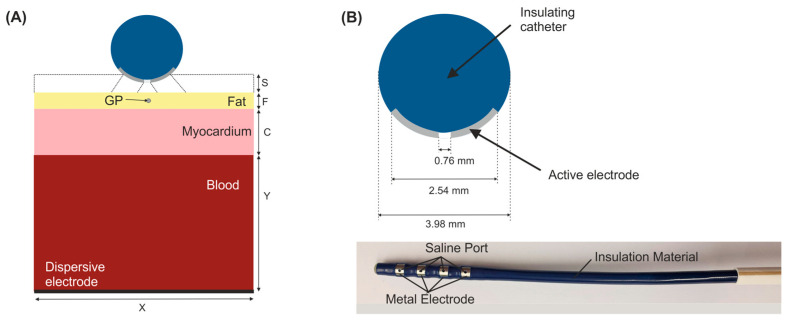
(**A**) Geometry of the 2D limited-domain model which only considers a fragment of the region of interest around the ablation device. Different layers were included below the ablation device: a saline layer (S = 0.5 mm) placed over an epicardial fat surface (F), myocardium (C = 2.7 mm) and blood layers. A ganglionated plexi (GP) with an area of 0.07 mm^2^ [4] is included in the centre of the epicardial fat layer below the ablation device. The dispersive pad is placed on the bottom surface of the model. (**B**) Detail of a circular section of the ablation device (AtriAN Medical, Galway, Ireland) with a hole in its centre for saline infusion [2,5].

We considered a 2D limited-domain model including only the region of interest around the ablation device. The validity of this approach has been previously demonstrated in comparison with a full torso model [5]. The model consisted of the ablation device placed over different layers (saline, fat, myocardium and blood) as is shown in Figure 1. We considered a circular section of the ablation device with the same dimensions as a real device [2,5]: a metal electrode 3.98 mm diameter, 2.56 mm length with a 0.76 mm diameter irrigation hole in its centre. The metal electrode of the ablation device was assumed to be initially inserted 0.25 mm in a saline layer of 0.5 mm thickness. This saline infusion between the ablation electrode and the epicardium fat (target site) acts as a ‘virtual electrode’, thereby ensuring the transmission of electrical energy to the target and preventing damage to the surrounding structures [2]. The fat layer varied between 0 (no fat) and 5 mm. Additional simulations included a ganglionated plexi (GP) in the centre just below of the irrigation hole of the ablation device with an area of 0.07 mm^2^ [4]. Myocardium thickness was of 2.7 mm [9] and the outer blood dimensions were similar to those of a limited-domain model of epicardial PEF ablation checked in a previous work by a sensitivity analysis, specifically X = 80 mm and Y = 40 mm [5].

### 2.2. Governing Equations

The model was based on an electrical problem, which was solved numerically by the Finite Element Method (FEM) with COMSOL Multiphysics (COMSOL, Burlington, MA, USA). A quasi-static approximation was employed to compute the electric field distribution. The transient cellular responses were not considered (i.e., membrane charging), then the electric field distribution can be computed by solving Maxwell’s equations in its Laplacian form [10]:

(1) ∇· (σ∇ϕ)=0

(2) E=−∇ϕ

(3) J=σE

where *σ* is the electrical conductivity of the material, *ϕ* the electrical voltage, ***E*** the electric field vector, and ***J*** the current density vector.

### 2.3. Material Properties

The electrical conductivity increases during pulse field delivery as the cell becomes more permeable to electrical current when PEF-induced pores are created. The change of the electrical conductivity during PEF ablation was modelled using a sigmoid function [11] dependent on the electric field magnitude as follows:

(4) $$\sigma \left(E\right)=\sigma_{0}+\frac{\sigma_{1}-\sigma_{0}}{1+10e^{-}\frac{\left(\left|E\right|-58000\right)}{3000}}$$

where σ_0_ and σ_1_ are the pre- and post-electroporation electric conductivities, respectively [5]. We considered the pre- and post-electroporation conductivities at 10 Hz and 500 kHz (σ_0_ and σ_1_, respectively) from IT’IS tissue database [12], as we checked in a previous sensitivity analysis [5] that the PEF-zone was identical for other low and high-frequencies. In this sense, the pre- and post- electrical conductivities were σ_0_ = 0.0537 S/m and σ_1_ = 0.0438 S/m for the epicardial fat, σ_0_ = 0.0275 S/m and σ_1_ = 0.152 S/m for the GP (assumed as grey matter), σ_0_ = 0.0537 S/m and σ_1_ = 0.281 S/m for the myocardium, and σ_0_ = 0.7 S/m and σ_1_ = 0.748 S/m for the blood. The electrical conductivity for the ablation electrode was the same as in [5] and for the saline was 1.392 S/m [13].

### 2.4. Boundary Conditions

Electrical boundary conditions were applied over the limits of the model. PEF ablation setting consisted of applying a pulse of 1000 V for 100 µs in monopolar configuration, i.e., between the metal electrodes of the ablation device and the dispersive pad [2,5]. To model this configuration, an electrical boundary condition of *ϕ* = 1000 V was applied at the metal active electrode, while *ϕ* = 0 V was set at the dispersive electrode. Electric current was set to be null in all the outer surfaces of the model except the surface corresponding to the dispersive electrode, which implied that the electric currents flow between the metal electrodes of the ablation device and the dispersive pad.

### 2.5. Outcome Analysis

Simulations were performed to assess the impact of different variables on the electric field distribution on the epicardial fat (target site) during epicardial PEF ablation. We used the 1000 V/cm isoline to assess and estimate ‘the PEF-zone’ size (maximum width and depth) as done in [7], since it was recently reported as the electric field threshold value for PEF-induced irreversible damage in the myocardium [4]. Firstly, we assessed the effect of having a saline layer present between the ablation electrode and the epicardium. We then evaluated the impact of the saline layer distribution on the epicardial fat surface (see Figure 1): the saline layer just below the irrigation hole of the ablation device, the saline extended over the entire surface of the ablation device, the saline extended over the entire surface of the epicardial fat with the ablation device embedded partially in it (0.25 mm), and the last case with the ablation device totally embedded in the saline layer. Once the best scenario regarding the saline characteristics was determined, we assessed the impact of the fat layer thickness. In this sense, we analysed the case without the presence of a fat layer and we then varied the fat layer thickness from 0.25 to 5 mm. We finally assessed the effect of having a GP within the epicardial fat, specifically in the centre of the epicardial fat just below the ablation device. To conduct this analysis we considered three fat layer thicknesses (1, 2 and 4 mm) and we compared these results to those obtained without having a GP.

## 3. Results

### 3.1. Impact of Saline Layer

**Figure 2:**
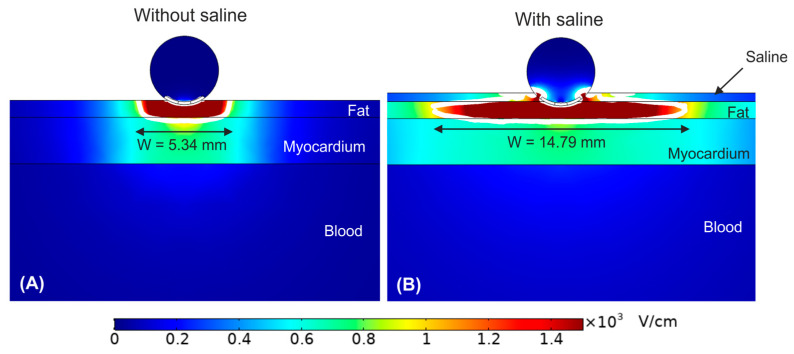
Electric field distribution around the target site (epicardium) for the case without (**A**) and with (**B**) a saline layer. The electrode is inserted 0.25 mm in the fat layer. The white contour corresponds to the 1000 V/cm electric field isoline.

**Figure 3:**
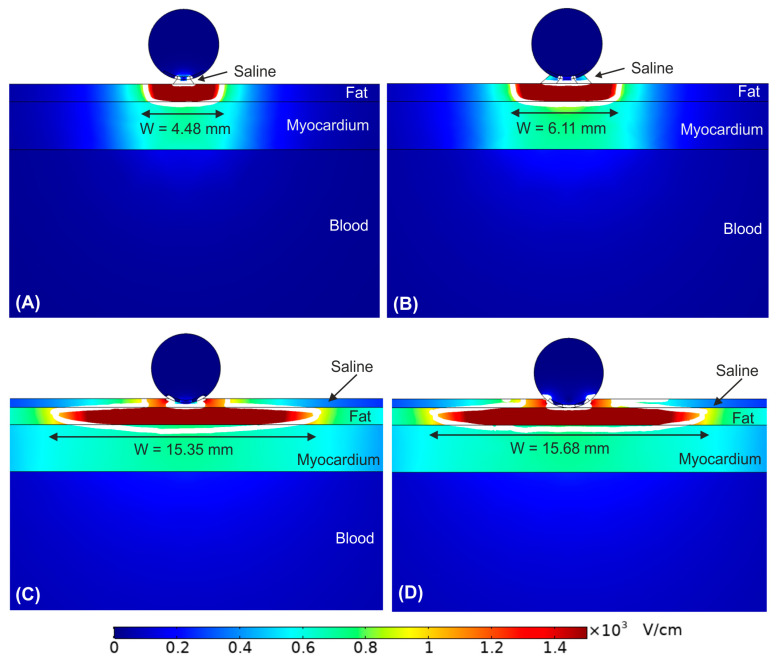
Electric field distribution around the target site (epicardium) to assess the effect of the extension of the saline on the epicardial fat. Different scenarios were compared: (**A**) the saline just below the irrigation hole, (**B**) the saline extends over the entire surface of the ablation device, (**C**) the saline extends over the entire surface of the epicardial fat with the ablation device embedded partially on it, and (**D**) the saline extends over the entire surface of the epicardial fat with the ablation device totally embedded in it. The white contour corresponds to the 1000 V/cm electric field isoline.

As shown in Figure 2, the PEF-zone (1000 V/cm isoline) was almost entirely in the epicardial fat (target), regardless of the presence of saline layer. However, the PEF-zone increased in width for the case with saline infusion, from 5.34 to 14.79 mm. While the results of Figure 2B corresponded with an infinite saline layer on the epicardial fat, it is also important to assess the impact of the saline distribution over the epicardial fat layer (target), which would be related with the irrigation flow and the relative position of the irrigation holes respect the tissue surface. In this regard, Figure 3 shows the electrical field distribution for the following cases: (A) a relatively small zone of saline infusion just below the irrigation hole, (B) occupying approximately the diameter of the ablation device, (C) saline extended over the entire surface of the epicardial fat with the ablation device embedded partially on the saline layer, and (D) the same saline distribution but with the ablation device totally embedded in the saline and touching the epicardium. We observed that the PEF-zone was always confined in the epicardium (target site) without damaging the underlying myocardium. As the saline section was extended, the PEF-zone width increased: from 4.48 mm with only a small saline zone just below the hole (Figure 3A) to 15.68 mm when the saline layer completely covering the epicardial fat with the ablation device totally embedded in it (Figure 3D). For this reason, we determined this last case was the best scenario to achieve a greater PEF-zone in the target, so we considered this configuration of saline for the rest of the simulations. However, it is also interesting to note that the PEF-zone was almost identical when the electrode was partially or fully inserted into the saline layer (Figure 3C,D).

### 3.2. Impact of the Epicardial Fat Thickness

**Figure 4:**
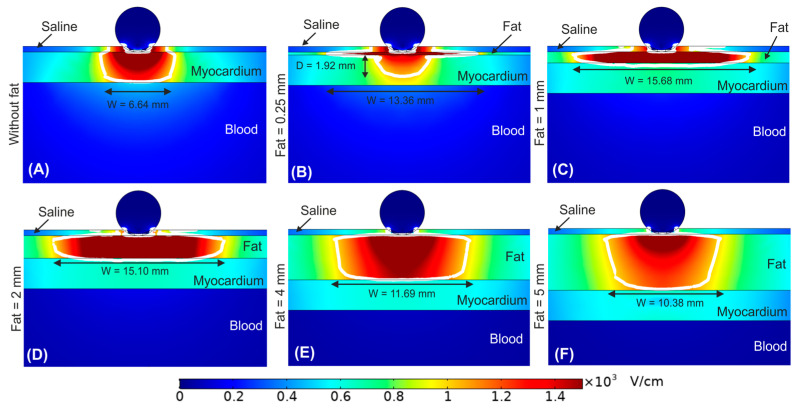
Electric field distribution around the target site (epicardium) to assess the effect of increasing the fat layer thickness. Different fat layer thicknesses were compared: (**A**) without fat layer, (**B**) 0.25 mm, (**C**) 1 mm, (**D**) 2 mm, (**E**) 4 mm, and (**F**) 5 mm fat layers. The ablation device is totally embedded in the saline layer. The white contour corresponds to the 1000 V/cm electric field isoline.

Figure 4 show the effect of increasing the fat layer thickness from 0 mm (no fat layer) to 5 mm. The fat layer was assumed to be homogeneous adipose tissue. We observed that as the fat layer is thicker, the electric field is mainly confined within it. However, in the case of very thin fat layer (<0.25 mm, Figure 4B), the electric field reaches high values within the myocardium, resulting in a transmural PEF-zone in case of no fat (Figure 4A). In contrast, for fat thickness ≥1 mm the PEF-zone was totally confined in the fat. Interestingly, as fat thickness increased, the PEF-zone width was reduced from the maximum size at 1 mm (15.68 mm, Figure 4C) to 10.38 mm at 5 mm fat layer (see Figure 4F).

### 3.3. Impact of the Ganglionated Plexi Embedded in the Fat

**Figure 5:**
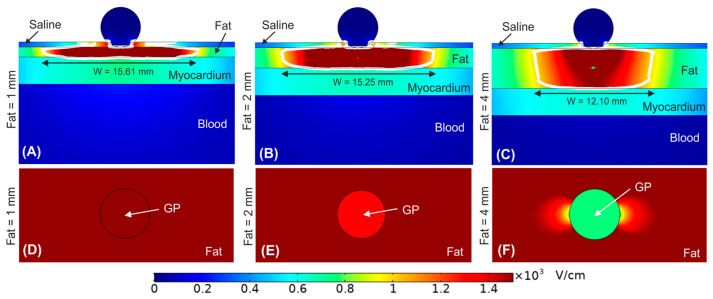
Electric field distribution around the target site (epicardium) to assess the effect of having a ganglionated plexi (GP) in the centre of the epicardial fat layer just below the ablation device. Different fat layer thicknesses were considered for comparison: (**A**) 1 mm, (**B**) 2 mm, and (**C**) 4 mm fat layers. Below is shown a magnified view of the GPs within the different fat layer thicknesses: (**D**) 1 mm, (**E**) 2 mm, and (**F**) 4 mm. The ablation device is totally embedded in the saline layer. The white contour corresponds to the 1000 V/cm electric field isoline.

Until now, the fat layer was considered a homogeneous adipose tissue. Figure 5 shows the effect of including a ganglionated plexi within the epicardial fat layer. In particular, we considered the GP positioned in the centre of the epicardial fat just below the irrigation hole of the ablation device for fat thicknesses of 1, 2 and 4 mm. Overall, we observed that the outer limits of the PEF-zone was not affected by the presence of a GP within the epicardial fat layer. For a 1 mm fat layer the PEF-zone width was 15.61 mm with GP vs. 15.68 mm without GP (Figure 5A vs. Figure 4C), whereas for fat layers with thickness ≥2 mm the PEF-zone width had an insignificant increment of 0.15 and 0.41 mm for 2 and 4 mm fat layers with GP, respectively (Figure 5B,C vs. Figure 4D,E). However, the results showed a marked distortion of the electric field around the GP itself for fat layers thickness ≥2 mm (Figure 5E,F), with a reduction of the electric field value just in the centre of the GP: 2251 V/cm, 1329 V/cm and 751 V/cm for fat thicknesses of 1, 2 and 4 mm, respectively.

## 4. Discussion

### 4.1. Main Findings

In this study, a computer model including the different types of tissue involved in the epicardial ablation of GPs was used to compute the electric field distribution and assess the efficacy of this technique under different conditions in terms of how much epicardial fat zone is ablated (i.e., values above 1000 V/cm) and to what extent the myocardium can be collaterally damaged. The advantage of computer modeling is that specific conditions related to irrigated saline, tissue thicknesses and presence of GPs can be independently controlled, which is complicated with an experimental setup. The main findings of the study are:

(1) In general, the PEF-zone is almost 100% circumscribed in the epicardial fat layer, with very little incidence in the myocardium (the 1000 V/cm isoline barely penetrates the myocardial layer).
(2) The presence of the saline on the epicardial fat causes the PEF-zone to spread laterally around the electrode, relatively independently of how embedded the electrode is in the saline layer.
(3) For a saline layer well spread over the tissue surface and an electrode fully embedded in the saline layer, the width of the PEF-zone decreases as the thickness of the fat layer increases. When the fat layer is very thin (<1 mm) the PEF-zone significantly penetrates the myocardial layer.
(4) The presence of a GP in the center of the fat layer hardly affects the size of the PEF-zone in the fat itself, but significantly alters the distribution of the electric field around the GP. In addition, the electric field values in the GP itself are significantly reduced as the fat layer increases.

This study has multiple clinical implications for PEF ablation of epicardial autonomic ganglia. The results show that the great difference in electrical conductivity between the fat (0.044 S/m) and the myocardium (0.281 S/m) in a layered structure such as occurs in epicardial ablation causes the greatest voltage drop to occur in the fat itself. In other words, the values of electric field are much higher in fat than in myocardium. In a previous modelling study on RF heating of subcutaneous tissue, we already observed how the high values of electric field (E-field) were concentrated mainly in the fat areas [14]. In that case, the fat was spatially distributed between septa of fibrous tissue (more conductive than fat). In contrast, in the epicardial PEF ablation, the fat is concentrated in a single layer that is spatially located between the ablation electrode and the myocardium. This greatly supports the therapeutic goal of causing electric field damage to the GPs (which are embedded in the fat layer) while preserving the myocardium.

When a zone of neuronal tissue (mimicking the GP) is included the results suggest that due to the lower electrical conductivity of the surrounding adipose tissue (0.0438 vs. 0.152 S/m), the E-field at the GP is reduced, and more markedly as the fat is thicker and consequently the GP is further away from the ablation electrode. This physical phenomenon was already observed in a computational modelling study of PEF in the context of the ablation of tumor nodules, which have a higher electrical conductivity than the surrounding healthy tissue [15]. This finding should be taken into account, as it could reduce the effectiveness of PEF ablation to achieve irreversible electroporation of GPs. According to a recent experimental study the irreversible electroporation damage threshold for neurons has been established at approximate E-field strength of 1200 V/cm [16]. Therefore, our simulations suggest that for fat layers thicker than 4 mm the GP may not be completely electroporated, since the E-field at the GP is 751 V/cm, much lower than that of the surrounding fat.

From a procedural point of view, our results confirm the usefulness of saline irrigation to create a ‘virtual electrode’ that ensures ‘electrical contact’ between the electrode and the epicardial fat, allowing the PEF-zone to spread laterally, increasing the probability of success of ablation in the case that the catheter is not positioned exactly over the target GP. In addition, the findings suggest that the saline layer must have sufficient extension, and not be limited to the area of the irrigation hole (see Figure 3). In fact, if the saline layer occupies only a very small area below the electrode (see Figure 3A), the width of the PEF-zone can become even slightly smaller (1 mm difference) than in the case that the saline layer is not present and a good contact between electrode and tissue is ensured (Figure 2A). Finally, despite the fact that the presence of the saline layer favors the creation of relatively wide PEF-zones, this effect is gradually reduced in the presence of thicker fat layers, especially from 4 mm (see Figure 4).

Note that the situation is different in both figures, since while in Figure 2A there is no saline and the electrode is inserted 0.25 mm into the fat, in Figure 3A the electrode is superficially in contact with a very small amount of saline, which hardly extends over the surface of the tissue. This results in a minimal electrical contact of the electrode with the tissue (only through the saline). This is possibly the reason for the difference of around 1 mm in lesion width between both cases. In any case, the conclusion is that these are not comparable situations because two factors are playing at the same time: amount of saline and electrode contact surface.

### 4.2. Limitations

The model was 2D, which implies that we assumed an infinitely long electrode. This means that the results only reflect the behaviour of the electric field right in the middle of the electrode, and not at its ends, where an edge effect could be found. Despite this, the results are relevant and have clinical implications because the model assumes that the electrode is properly positioned exactly on the target, i.e., that the midpoint of the electrode is on the position of the GP to be ablated. The geometry of the GP was simplified to be a circle with the same mean area as observed in histological images of GPs in humans [4] within the epicardial fat layer. However, this is not an accurate geometry of GPs, which are really dispersed as randomly clusters of neuronal cells with thin connective tissue shell around these clusters. In fact, the precise anatomical structure of GP varies considerably between humans [17]. As the electrical conductivity of the GP is not currently characterised, we assumed properties of grey matter. In addition, our model assumed values of pre- and post-electroporation electrical conductivities based on values for different frequencies (low and high, respectively). Although this is common practice in computational models of electroporation, and moreover makes sense from a physical point of view, our results should be viewed with caution until specific experimental measurements of these electrical conductivities are available under the same conditions that occur in PEF epicardial ablation and for each involved specific tissue. For all of this, our results could be slightly different taking the accurate structure and properties of GPs in the target into account.

Finally, our model ignored both endothelium and epithelium, i.e., the single layers lining the inner and outer surface of the heart wall. They are single-cell layers supported by a lamina propria. In terms of computer modelling of ablative therapies, and as far as we know, they have never been included in the geometry of the model, probably for two reasons: (1) Their electrical properties may not differ greatly from the characteristics of the tissues they surround (myocytes, fat, vessels, blood, conduction system), and (2) their extremely small thickness possibly has little impact on electrical field distributions.

## 5. Conclusions

Our results confirm the usefulness of the irrigated saline layer to achieve a greater width of the area affected by PEF. The presence of epicardial fat between the electrode and the myocardium favours the high electric field values being circumscribed to the fat layer, hardly affecting the myocardium. The greater electrical conductivity of neuronal tissue compared to adipose tissue causes the electric field to be distorted in the surroundings of the GPs, and the values are lower than in the surrounding fatty tissue.

## Data Availability

Not applicable.
